# Assessment and analysis of accents in air traffic control speech: a fusion of deep learning and information theory

**DOI:** 10.3389/fnbot.2024.1360094

**Published:** 2024-03-05

**Authors:** Weijun Pan, Jian Zhang, Yumei Zhang, Peiyuan Jiang, Shuai Han

**Affiliations:** ^1^Air Traffic Control Automation Laboratory, College of Air Traffic Management, Civil Aviation Flight University of China, Guanghan, China; ^2^Department of Operational Supervision, Operation Supervisory Center of CAAC, Beijing, China

**Keywords:** air traffic control speech, data quality, accent disruption, quantitative speech evaluation, speech evaluation optimization, speech recognition impact

## Abstract

**Introduction:**

Enhancing the generalization and reliability of speech recognition models in the field of air traffic control (ATC) is a challenging task. This is due to the limited storage, difficulty in acquisition, and high labeling costs of ATC speech data, which may result in data sample bias and class imbalance, leading to uncertainty and inaccuracy in speech recognition results. This study investigates a method for assessing the quality of ATC speech based on accents. Different combinations of data quality categories are selected according to the requirements of different model application scenarios to address the aforementioned issues effectively.

**Methods:**

The impact of accents on the performance of speech recognition models is analyzed, and a fusion feature phoneme recognition model based on prior text information is constructed to identify phonemes of speech uttered by speakers. This model includes an audio encoding module, a prior text encoding module, a feature fusion module, and fully connected layers. The model takes speech and its corresponding prior text as input and outputs a predicted phoneme sequence of the speech. The model recognizes accented speech as phonemes that do not match the transcribed phoneme sequence of the actual speech text and quantitatively evaluates the accents in ATC communication by calculating the differences between the recognized phoneme sequence and the transcribed phoneme sequence of the actual speech text. Additionally, different levels of accents are input into different types of speech recognition models to analyze and compare the recognition accuracy of the models.

**Result:**

Experimental results show that, under the same experimental conditions, the highest impact of different levels of accents on speech recognition accuracy in ATC communication is 26.37%.

**Discussion:**

This further demonstrates that accents affect the accuracy of speech recognition models in ATC communication and can be considered as one of the metrics for evaluating the quality of ATC speech.

## 1 Introduction

Due to the scarcity, difficulty in acquisition, and high cost of labeling of air traffic control (ATC) speech data in various control scenarios, ATC speech recognition models are prone to data sample bias and class imbalance issues during model training, directly affecting the recognition accuracy of speech recognition models. This situation may further lead to incorrect aircraft control decisions made by other ATC systems that rely on recognizing text as input, posing significant flight safety risks and potential hazards.

To address these issues, this study delves into data quality and constructs a comprehensive data ecosystem (Downs et al., 2021). By using a quantitative approach to quantify accents, the quality of speech data is calibrated. Subsequently, different strategies for combining data quality categories are selected according to the requirements of different model application scenarios, ensuring that the trained speech recognition models achieve optimal recognition accuracy. This initiative plays a crucial foundational role in advancing the integration, application, and decision-making of civil aviation intelligence, and is expected to promote the civil aviation air traffic management intelligence to a higher level.

As is well-known, deep learning models are inherently sensitive to data distribution due to their nature of self-supervised learning (Pan et al., 2023). However, dealing with incomplete instances is a common phenomenon when processing real-world datasets (Liu and Letchmunan, 2024). Typically, to ensure the completeness of data collection, methods such as fuzzy clustering, interpolation, multisensory information fusion, and similarity measurement are employed during data preprocessing to fill in missing data and improve the performance of machine learning (Choudhury and Pal, 2022; Liu, 2023, 2024). However, in some special fields, simulating missing data can become exceptionally cumbersome, or the supplemented missing data may differ significantly from real data. Therefore, the approach adopted in this paper is to fully leverage the value of the collected real data and deeply explore its data worth, avoiding the complexity of simulating missing data while ensuring the authenticity of the entire dataset and avoiding the use of synthetic data. Currently, there are two main methods for speech quality assessment. One is non-intrusive black-box models, such as Mean Opinion Score (MOS) (International Telecommunication Union, 1996), which are artificial fuzzy speech quality evaluation methods. According to the International Civil Aviation Organization (ICAO) English Language Proficiency Standard (ICAO Annex 1, Personnel Licensing), the MOS evaluation specification for ATC speech quality is shown in Table 1, which classifies the ATC speech quality into levels 1 to 5. Level 5 is the best quality. Although they can assess speech quality, they cannot deeply understand the internal logic of speech quality evaluation within the model. Another method is to use the Perceptual Evaluation of Speech Quality (PESQ) algorithm (International Telecommunication Union, 2001), but it requires standard pronunciation samples as references, making it difficult to deploy and unsuitable for complex and variable scenarios.

**Table 1:** The MOS evaluation specification for ATC speech quality (Levels 1–5, Level 5 is the best quality).

**Level**	**Evaluation standards**
1	Standard ATC communication have less content coverage; too fast or too slow speech; complex grammatical structures and sentence patterns; pronunciation, stress, rhythm and intonation influenced by first language or regional variations; speech containing words that can mislead semantic understanding; Higher interference from electromagnetism, ambient noise, etc;
2	Standard ATC communication have limited coverage and are spoken too fast or too slow, with a small amount of natural language and complex grammatical structures and sentence patterns; pronunciation, stress, rhythm, and intonation rarely interfere with the course of the communication, although they are affected by first-language or regional variations; and there are isolated instances of speech that mislead semantic comprehension of vocabulary;
3	Standard ATC communication coverage meets general requirements with normal speech rate and regular air traffic control content; communication system speech signals occasionally stutter;
4	Standard ATC communication cover a wide range of basic grammatical structures and sentence patterns; normal speed of speech, fluent speech, with isolated misdirected semantic comprehension of vocabulary;
5	Standard ATC communication cover a wide range of topics, with structured speech content, fluent speech, and no speech that would mislead semantic understanding.

### 1.1 Background of the proposed ATC speech accent evaluation metric

**Figure 1:**
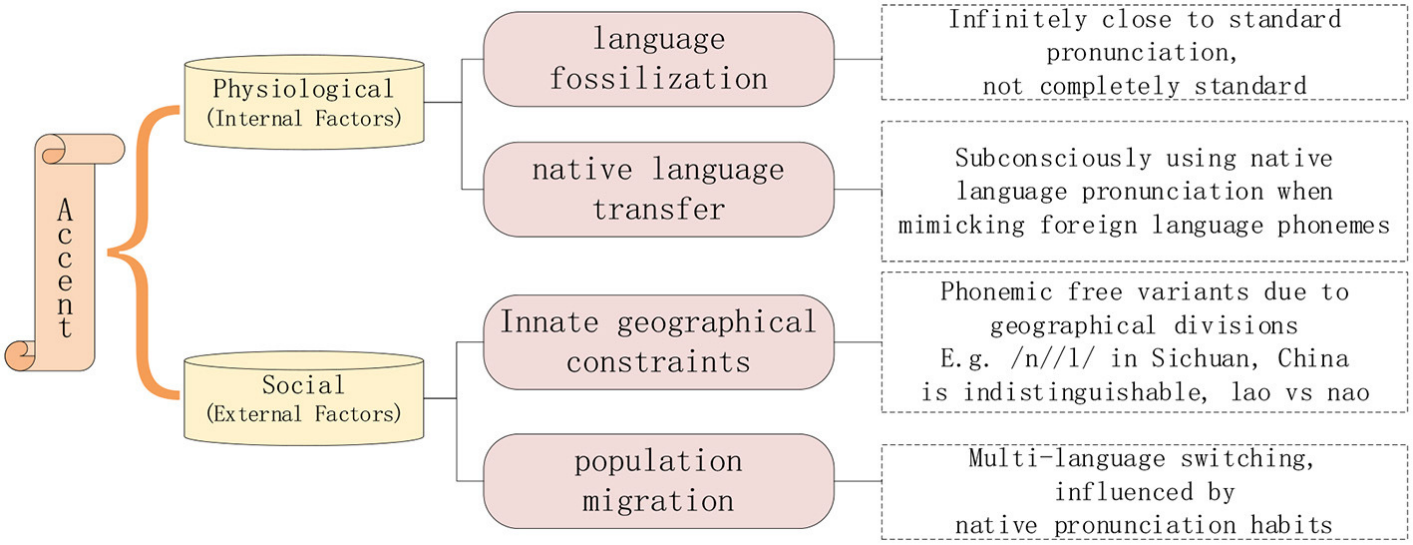
Causes of the micro-accent phenomenon.

The differences between ATC speech and everyday conversation speech lie in their rapid pace, unique pronunciation rules, complex noise background, and the phenomenon of multilingual switching and accents. In the actual air traffic control communication process, although the ICAO in the “Manual on the Implementation of ICAO Language Proficiency Requirements” (International Civil Aviation Organization, 2009) stipulates that civil aviation frontline workers must have a language proficiency of at least level four. That is, they must maintain a standard pronunciation while working, which does not affect the understanding of semantic content. However, everyone has an accent, and the reason for the accent is shown in Figure 1, which is only avoided in the frontline work of civil aviation, so the degree of accent is slightly weak, called “micro-accent phenomenon”.

As shown in the Table 2, accents may lead to distortion of speech signals, making it difficult for conventional speech recognition models to accurately match accent variants, involving multiple aspects such as acoustic models, language models, and encoder-decoders, thereby affecting the performance of speech recognition models. The ATC speech recognition model is required as an intelligent application to assist in improving efficiency in intelligent civil aviation. Although it wants to try its best to mimic the human mind to achieve a specific intelligent task, it is still essentially a class of machine learning models, and their recognition accuracy in practice may be affected if the various accents and speech variants are not covered in their previous cognitive learning. Because of this, the preprocessing of speech data quality assessment in speech recognition models is very important, making full use of the existing historical data to evaluate the data quality, so that the training and testing sets of speech recognition models are distributed evenly, and the various accents and speech variants are covered extremely well, so as to improve the recognition performance of the models.

**Table 2:** The impact of accents on different speech recognition models.

**Model classification**	**Model characteristics**	**Models composition**	**Impacts**
Traditional speech recognition models	Training and optimizing the acoustic model and language model separately	Acoustical model	Changes in acoustic features
		Language model	Lead to changes in vocabulary usage and order, making it more challenging for language models to understand.
End-to-end speech recognition models	From acoustic features to integrated text	Encoder-decoder	The degradation of speech recognition generalization and adaptability

### 1.2 Related work

ATC speech accent assessment, essentially accent pronunciation segment perception, detects the phonemes in the speaker's speech, and compares the detected speech phonemes with the phoneme sequences transcribed from the speech text. If the comparison results are consistent, the pronunciation is standard; if they are not, an accent is present. As shown in Table 3, the methods for evaluating speech quality are evolving from traditional techniques to deep learning, with continuous refinement and improvement. In earlier phoneme detection methods, scholars used an “acoustic + anatomical” mechanism to map the target phonemes to corresponding phonetic features in speech. This involves assessing the size/shape of the resonator and considering whether there are obstacles to articulation, and ultimately identifying specific phonemes (Tepperman and Narayanan, 2008). Although this method is relatively high in accuracy, it is overly dependent on the expertise reserves of domain experts, and the research base is too high, which limits its wide application. With the development of machine learning and deep learning technology, a series of mutually integrated and optimized Goodness of Pronunciation (GOP) models based on Hidden Markov Model (HMM) and Gaussian Mixture Model (GMM) have come into view (Witt, 2000; Kanters et al., 2009; Sudhakara et al., 2019). These models are usually structured in two parts, the first phase is acoustic feature extraction as input to the algorithm, the second phase is the calculation of the probability of occurrence of each phoneme in each time frame (i.e., a posteriori probability) on a given sequence of acoustic feature observations, and the likelihood of comparing the sequence of phonemes from a real text transcription with the posterior probability as an assessment of the goodness of pronunciation (Huang et al., 2017). However, most of the improved models of such methods focus on the optimization of the a posteriori probability calculation method in the second stage, which has great limitations and only focuses on the processing of speech acoustic features, which cannot cover the comprehensive information of speech well, and will miss the important speech information such as frequency, intonation, and rhythm of speech. As the development of deep learning models becomes more and more mature, people begin to introduce more comprehensive speech information features, such as Mel-frequency cepstral coefficient (MFCC) and filter bank cepstral coefficient (FBANK). The PESQ algorithm is used to compare the difference between the speech signal and the reference speech signal to make an objective evaluation of speech quality (Lee and Glass, 2012; Lee et al., 2013, 2016), but the algorithm requires standard pronunciation samples as a control, which leads to limitations in land and air call recognition. It is only suitable for the evaluation of speech pronunciation quality with fixed text content, and it is more difficult to be deployed for the complex and changing control scenarios and the unfixed text content of control instructions in air traffic control. In addition, there are researchers who indirectly identify phonemes with the help of speech recognition models by recognizing speech signals as text, and text is converted into corresponding phonemes (Chan et al., 2015; Chorowski et al., 2015; Watanabe et al., 2017), but this approach may introduce the continuous accumulation of errors in the automatic speech recognition (ASR) model, which affects the correctness and reliability.

**Table 3:** Evolution of speech quality evaluate methods: from traditional to deep learning.

**Classification of methods**		**Methodological characteristics**	**Advantages**	**disadvantages**
Traditional non-deep learning methods		Adoption of the “Acoustic + Anatomical” mechanism	Relatively high precision; Highly interpretable	Over-reliance on expertise and high research base; Limits wide range of applications.
Traditional machine learning methods		Staged and able to adjust errors in a timely manner	Staged assessment with low error	Inability to fully cover speech information
Deep Learning Methods	Based on speech signal processing	Introduction of more comprehensive speech information features	More comprehensive speech information	Standard pronunciation control samples are required, which is limiting
	Based on speech recognition models	Converting speech signals to text and then to phonemes	Extracting phonemes directly from speech	Introduce errors into the automatic speech recognition model and continue to accumulate

Therefore, this paper integrates the multiple advantages of the above methods and establishes a phoneme recognition model based on the fusion of speech and sentence a priori textual features by using the mutual integration of deep neural networks and information theory. The introduction of contextual context and attention mechanism makes it possible to capture speech information more comprehensively, thus improving the accuracy of the phoneme recognition model. The main purpose of this paper is to propose an objective quantitative metric for analyzing the quality of ATC speech—accent. The aim is to elucidate whether ATC speech classified according to this metric has an impact on the performance of speech recognition models and the relationship between the interactions.

### 1.3 Structure of the paper

This paper is divided into five parts: the first chapter describes the background, reasons, and relevant arguments for using “accent” as an evaluation metric for ATC speech quality. In this way, it explains the necessity of accent evaluation and analyses and summarizes the research methods used and challenges faced by other experts and scholars. In Chapter 2, the technical methodology and model used to adopt 'accent' as an evaluation metric of ATC speech quality are described. Chapter 3 details the experimental processes and results. In Chapter 4, the ATC speech data evaluated based on the above evaluation methods will be applied to the speech recognition model, using correlation coefficients and comparison experiments to verify the validity of the effect of different ATC speech accent levels on the recognition results. Chapter 5 summarizes the main contents of the whole paper and explains the application value and significance of the research results.

## 2 Technical methods and modeling

### 2.1 Technical route analysis

In this paper, we are inspired by the Computer Assisted Pronunciation Training (CAPT) research method (Feng et al., 2020), which adopts the training method of one speech corresponding to multiple texts, and locates the mispronounced pronunciation segments by introducing multiple different texts corresponding to the same speech. While the purpose of this study is to detect the presence of accents, which also belongs to a kind of pronunciation error, but unlike the CAPT method it is difficult to artificially annotate the sequence of ATC speech phonemes, and the experimental conditions we already have can only be to annotate the correct text it corresponds to. Therefore, we adopt the training method of multiple speech corresponding to one text, so that one text corresponds to multiple different speech, so that the model can be better generalized to the pronunciation characteristics of ATC speech, and improve the accuracy of the prediction of the phoneme recognition model.

**Figure 2:**
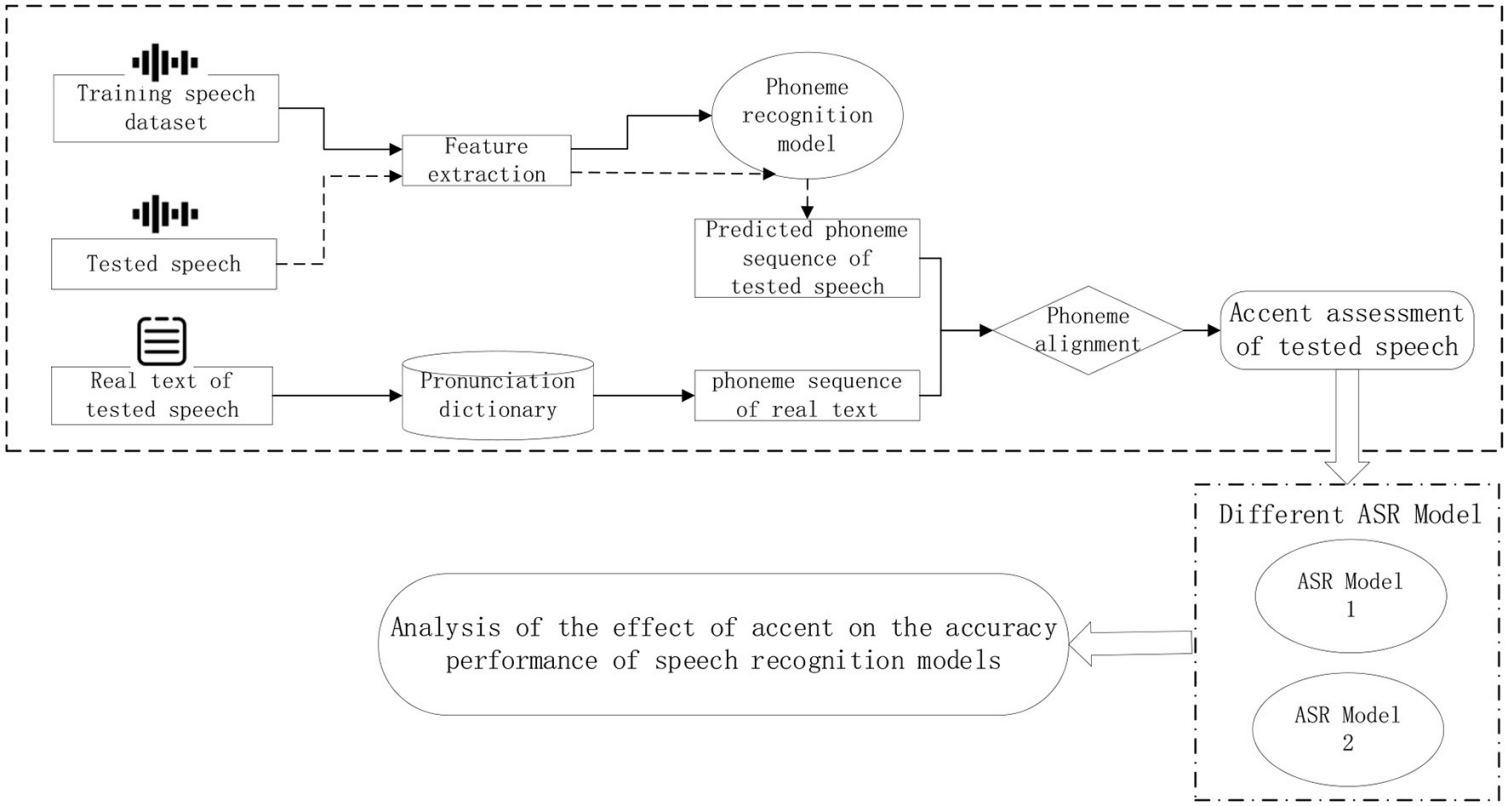
Overall research ideas and technical routes.

Therefore, the technical route designed in this paper is shown in Figure 2, which uses a publicly available standard acoustic-phoneme database defaulting to its non-accented dataset, to build a standard speech phoneme database and use it as a training set, and secondly, the training set and incorporate a certain amount of ATC speech data, so as to make the model adapt to the pronunciation characteristics of ATC. The phoneme recognition model is trained using the assembled pronunciation data, and different speech features are mapped to the corresponding phonemes. The speech to be tested is passed through the trained phoneme recognition model to predict the sequence of phonemes contained in the speech to be tested, and then this sequence is compared with the sequence of phonemes corresponding to the correct text transcribed from the speech to be tested. If an accent is present in the speech to be tested, the model may recognize the speech segment as other phoneme variants. Therefore, the recognized phoneme sequences are compared with the phoneme sequences transcribed from the speech text, and where there are differences, it is assumed that an accent fragment is present.

### 2.2 Model architecture

**Figure 3:**
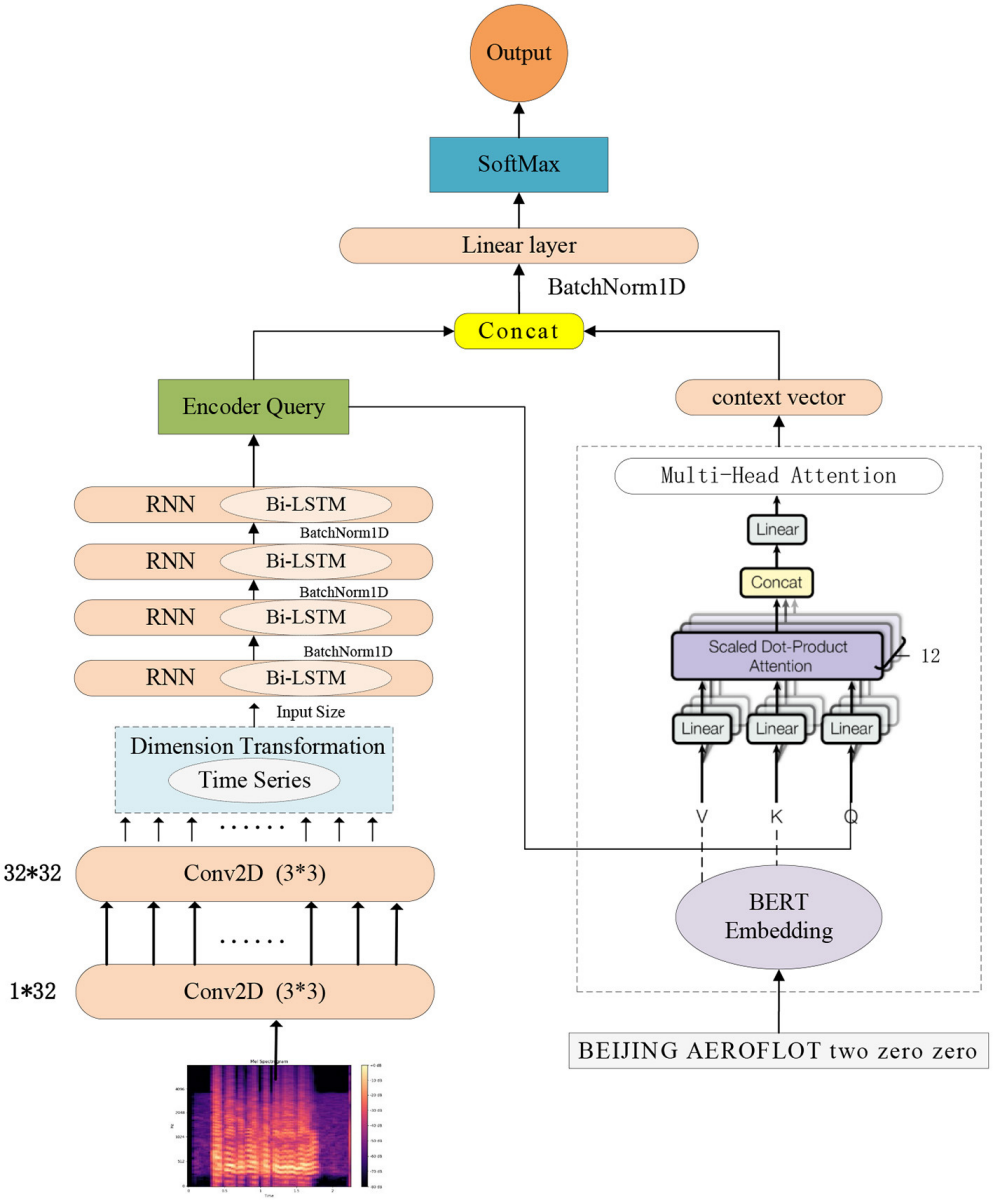
Structure of the phoneme recognition model.

The overall architecture of the model consists of an audio coding module, a priori text coding module, a feature fusion module, and a full connectivity layer, as shown in Figure 3. The input of the model is the speech and the corresponding a priori text, and the output is the phoneme sequence of the speech.

The total number of frames of the whole speech is **T**, the speech feature vector ***X* = [*x***_**1**_**;*x***_**2**_**;...;*x***_***t***_**;...;*x***_***T***_**]**, where ***x***_***t***_ represents the feature vector of the speech at the t-th frame. The audio encoding module consists of two two-dimensional (2D) convolutional layers and four bidirectional long short-term memory (Bi-LSTM) layers. Batch normalization is applied to the input of each bidirectional LSTM to mitigate the vanishing gradient problem, accelerate convergence, and enhance the model's robustness. The specific formula for batch normalization is shown in Equation (1).


(1) $$\begin{array}{l} \begin{array}{c} {\hat{x}}_{k}^{\left(t\right)}\;=\;\frac{x_{k}^{\left(t\right)}\;-\;E\left[x_{k}\right]}{\sqrt{Var\left[x_{k}\right]}} \end{array} \end{array}$$

$x_{k}^{(t)}$ represents the observed value of the k-th dimension of the t-th frame's speech feature vector. ${\hat{x}}_{k}^{(t)}$ represents observations after batch normalization. ***E*[*x***_***k***_**]** is the mean of the eigenvalues of all samples in the current batch on that dimension. $\sqrt{Var[x_{k}]}$ is the variance of the eigenvalues of all samples in the current batch on that dimension.

**Figure 4:**
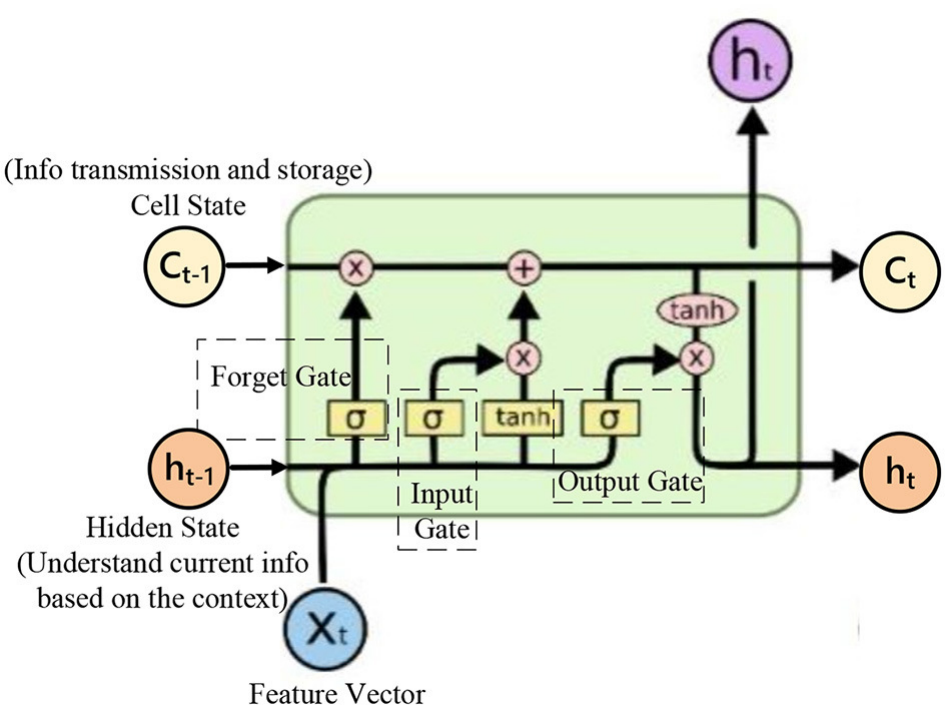
A diagram of an LSTM structure.

Speech data possesses strong temporal characteristics and rich time-frequency information. Introducing Bi-LSTM into the audio encoding module allows for comprehensive coverage of speech information. As shown in Figure 4, it illustrates a diagram of an LSTM structure. Bi-LSTM propagates information in both forward and backward directions, considering past and future information simultaneously, resulting in more accurate feature extraction of audio data. Bi-LSTM utilizes internal gating mechanisms to finely control the flow of information. The role of the forget gate is to enable the network to maintain appropriate memory between different speech segments, facilitating a better understanding of long-term speech patterns. Meanwhile, the input gate is responsible for dynamically incorporating new input information and updating the cell state. These intricate designs enhance Bi-LSTM's performance in modeling temporal information. The input speech features are passed through the audio coding module to obtain the output feature sequence denoted as ***Encoder*** ***Query***, which is subsequently used for the computation of the attention vector and the feature fusion operation. The calculation of ***Encoder*** ***Query*** is shown in Equation (2).


(2) $$\begin{array}{l} Encoder\;Query\;=\;CNN-RNN\left(X\right) \end{array}$$

The a priori text encoder corresponding to speech uses the Bidirectional Encoder Representations from Transformers (BERT) model, which is a bidirectional Transformer encoder capable of efficiently extracting features of the input text from both directions. The text encoder input is the sequence ***P* = [*p***_**1**_**, ..., *p***_***n***_**, ..., *p***_***N***_**]** of phonemes corresponding to the a priori text, ***p***_***n***_ is the phoneme at the n-th position, and the length of the phoneme sequence is **N**.

The output sequence features of the phoneme sequence **P** after BERT coding are used as sequence **K** and sequence **V**, respectively, where ***K*=*V***, and are input to ***Mlti*−*attention*** together with ***Encoder*** ***Query***. The purpose of the multi-head attention mechanism calculation is to assist the training of the audio coding module to accelerate the alignment and improve the accuracy, which is shown in Equations (3)–(5).


(3) $$\begin{array}{l} attention\left(Q,K,V\right)\;=\;softmax\left(\frac{{QK}^{T}}{\sqrt{d_{K}}}\right)V \end{array}$$

(4) $$\begin{array}{l} Multi-Head\left(Q,K,V\right)\;=\;concat\left({head}_{1},{head}_{2},\ldots ,{head}_{h}\right)W^{0} \end{array}$$

(5) $$\begin{array}{l} {head}_{i}\;=\;attention\left(QW_{i}^{Q},KW_{i}^{K},VW_{i}^{V}\right) \end{array}$$

The features produced by each attention head are weighted and summed to form a new feature vector, denoted as **context** **vector**. The previous acoustic features are used to make acoustic residuals to reduce the model error. A beam search is performed to predict the phoneme sequence $P\prime =[p_{1}^{\prime },...,p_{n}^{\prime },...,p_{N}^{\prime }]$. The results predicted for each frame phoneme are shown in Equation (6).


(6) $$\begin{array}{l} p_{t}^{{\;}^{\prime }}\;=\;softmax\left(W\left(contextvector⊕Q\right)+b\right) \end{array}$$

In this equation, **⊕** represents the concatenation of two vectors, and $p_{t}^{{\;}^{\prime }}$ represents the predicted phoneme for the t-th frame.

## 3 Experimental results and validation

### 3.1 ATC Speech data collection

This experiment focuses on evaluating the degree of accent in ATC speech, and observing and measuring the effect of ATC speech with different degrees of accent on the recognition accuracy performance of speech recognition models. According to a research study (Jahchan et al., 2021), multi-language inter-switching tends to produce more pronounced accents than geographic switching, and individual languages have their own pronunciation habits. For example, civil aviation pilots in various countries are generally familiar with the English pronunciation of the destination country in advance before executing international flights, so that they can quickly make corresponding feedback in the first time after receiving the speech control instructions to ensure the absolute safety of aviation operations (Romero-Rivas et al., 2015).

Therefore, the dataset used for model pre-training consists of the TIMIT database (Garofolo, 1993), which is an acoustic-phoneme continuous speech corpus created by Texas Instruments and Massachusetts Institute of Technology. The TIMIT dataset has a speech sampling frequency of 16 kHz and contains a total of 6,300 sentences, all of which are manually segmented and labeled at the phone level. This paper focuses on accents caused by multilingual switching in different national contexts, so the TIMIT dataset, with its distinctive feature of phone level labeling for each speech sample, is ideal for setting up a standard pronunciation database.

To ensure the professionalism of the data, our study covers 20,000 pieces of ATC speech data, of which 15,000 are from the first-line approach control recordings of the East China Air Traffic Control Bureau of the Civil Aviation Administration of China (CAAC), and the other 5,000 are from the recordings on the control simulators, with a total length of about 30 h. The 5,000 on-simulator recordings were collected for the purpose of model training for the adaptation of pronunciation characteristics of ATC speech, so we purposely looked for experienced controllers to avoid accents when collecting this part of the data. Notably, we use these 5,000 control simulator recordings as the standard pronunciation to ensure high accuracy of the trained speech model. Finally, a batch of the first-line approach control recordings was selected from the dataset for phoneme sequence recognition by the phoneme recognition model, so as to calculate the degree of accent of the speech data. In the experiments, the speech data were classified into different accent levels according to the distribution of the accent degree of the speech data. In addition, in order to observe the influence of different ATC speech accent degrees on the correct rate of speech recognition model recognition, this paper also adds two speech recognition models for comparative verification experiments (PPASR ASR model and Wishper ASR model, respectively), and the two speech recognition models used still pick the same ATC speech data from them for fine-tuning training, and then analyze the relationship between the influence of ATC speech accents on the recognition accuracy of speech recognition model performance.

The experiments were conducted on a Linux operating system with the following computer configuration: an Intel Core i5-8400 processor, 56G of running memory, an NVIDIA RTX4090 24G graphics card, a 250 GB solid-state drive, and a 3.6 TB hard disk drive Speech feature extraction was performed using the Kaldi toolkit (Povey et al., 2011) to extract high-quality acoustic features from the raw speech signal for the flow of information between modules in the subsequent model.

### 3.2 Speech data preprocessing

**Figure 5:**
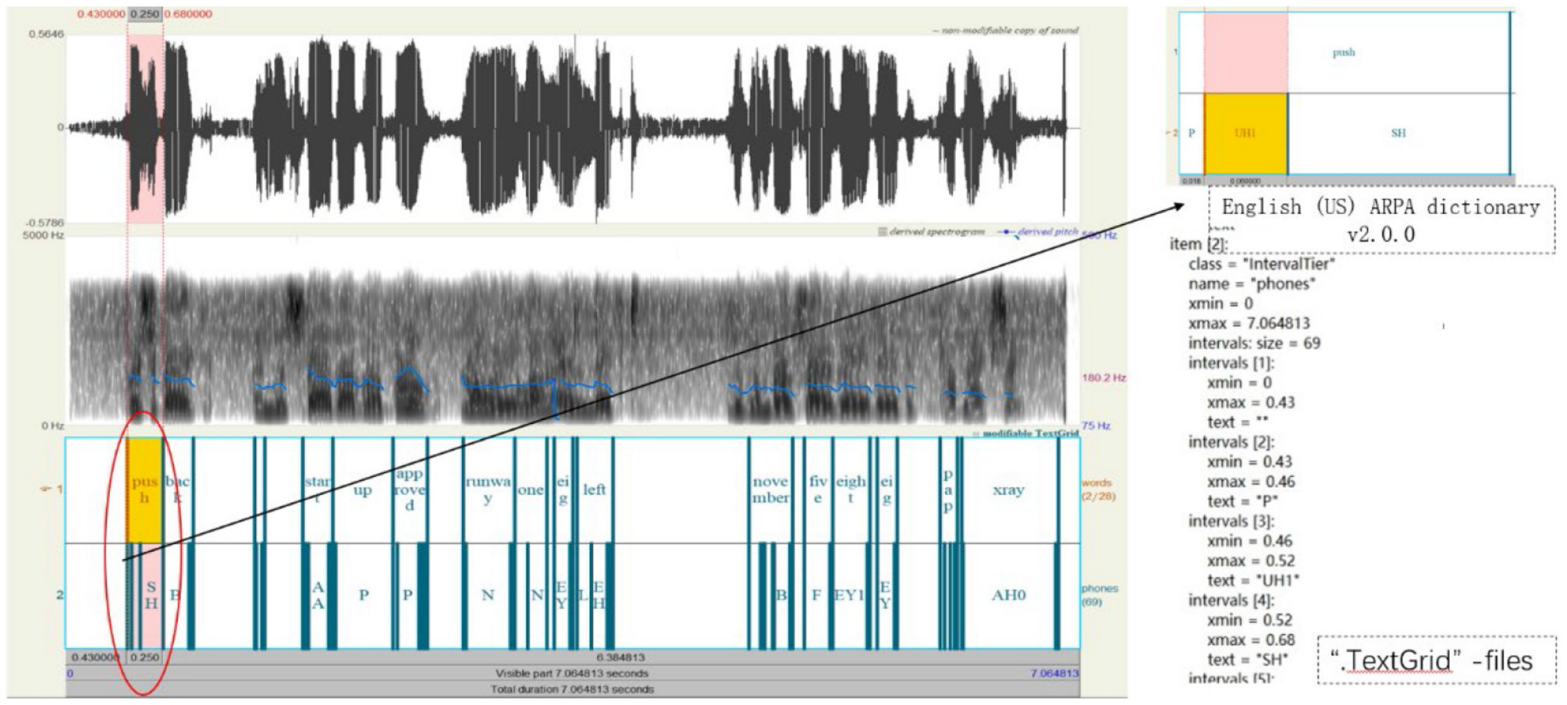
Data preprocessing process diagram (Control instructions: push back and start up ap-proved runway one eight left NOVEMBER five eight eight PAPA XRAY).

The Montreal Forced Aligner (MFA) phoneme alignment toolkit (McAuliffe et al., 2017) is used to align the speech segments in speech data with their corresponding text, generating “.TextGrid” files. These files contain time markers at the phoneme level and provide information about their positions within the speech. Additionally, the aligning results can be visualized using Praat software (Styler, 2013), as shown in Figure 5, facilitating subsequent speech processing tasks.

### 3.3 Phoneme recognition model training results

The details of the architecture are shown in Table 4. The phoneme sequence prediction (decoding) performance is evaluated using the decoder.wer() function, which calculates the word error rate (***WER***_***Phoneme***_***ASR***) between the output of the phoneme recognition system and the real text phoneme sequence, as shown in Equation (7). That is, how many insertion, deletion and substitution operations need to be performed to convert the phoneme sequence output by the model to the original text phoneme sequence. The lower the value of _***Phoneme***_***ASR***, the better it is, indicating the better phoneme sequence recognition performance of the model.


(7) $$\begin{array}{l} WER\text{\_}Phoneme\text{\_}ASR\;=\;\frac{S+D+I}{N} \end{array}$$

**Table 4:** Details of architecture.

**Model structure**	**Input size**	**Output size**	**Parameter setup**
Conv2D 1	(32, 80)	(32, 40)	kernel_size = (3, 3), stride = (1, 2), padding = (1, 1)
Conv2D 2	(32, 40)	(32, 20)	kernel_size = (3, 3), stride = (2, 2), padding = (1, 1)
LSTM	(32, 20,1)	(32, 768)	hidden_size = 384, bidirectional = True
Bert embedding	(32, 50)	(32, 50, 768)	Batch_size = 32, Max_length = 50
Multi-head attention	(32, 20, 768), (32, 50, 768)	(32, 768)	num_heads = 12, in_features = 768, out_features = 768
Concat	(32, 768), (32, 768)	(32, 1536)	dimension = −1
Linear layer	(32, 1536)	(32, 39)	Num_class = 39

**Figure 6:**
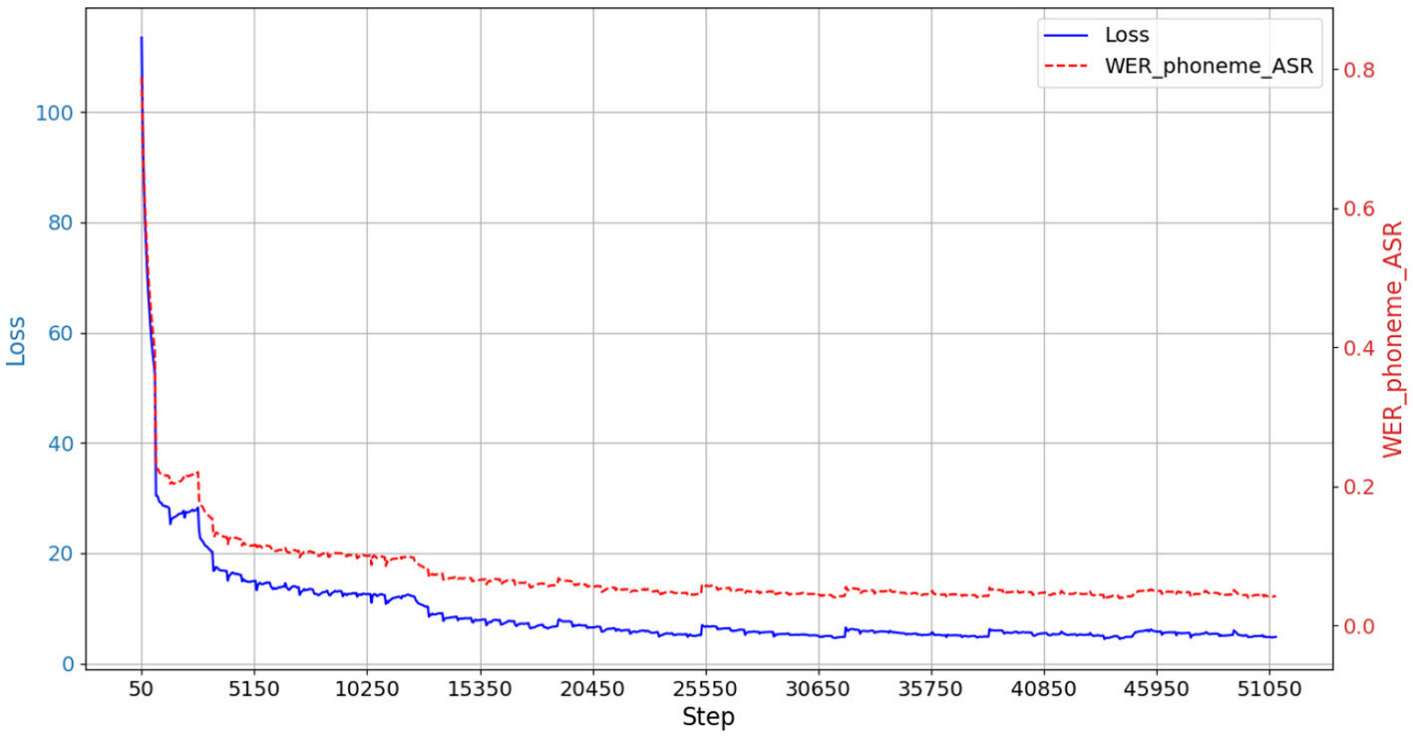
Visual monitoring of the training process.

Where **S** denotes the number of substitutions, **D** denotes the number of deletions, **I** denotes the number of insertions, and **N** denotes the number of real text phoneme sequences. After testing of the model, according to the statistical analysis results the phoneme recognition error rate (***WER***_***Phoneme***_***ASR***) of the model is 15.65%. The model training process is shown in Figure 6. In Figure 6, the vertical axes reflect the strengths and weaknesses of the model performance. The left and right vertical axes are mainly different in the unit of measurement, and the Loss value is a continuous, scalar value that measures the difference between the model's predicted value and the true value. A smaller Loss value indicates better model performance. ***WER***_***Phoneme***_***ASR*** is a discrete percentage indicating the percentage of difference between the predicted and true values of the model used as a metric.

### 3.4 ATC speech accent level classification

The overall framework designed in this paper is aimed at assessing the impact of accent on the performance of a speech recognition model for ATC speech. When the recognized phonemes in the test speech differ from the phonemes corresponding to the real speech text transcription, it indicates an acoustic difference between the pronunciation of the phoneme and the standard pronunciation, resulting in a slight accent phenomenon. We measure the degree of accent in the speech by evaluating the difference between the phoneme recognition sequence of the test audio and the real sequence (i.e., the phoneme recognition error rate). The error rate computed here is for the recognized and true phoneme sequences of the speech to be tested, i.e., it is the computed measure of the degree of accent of the ATC speech as proposed in this paper, denoted by ***WER***_***Accent***. The principle of calculating ***WER***_***Accent*** is consistent with Equation (7), but due to the characteristics of ATC speech itself, such as fast speech speed and noise interference, there are some overly distorted results in the experimental results, and the number of phonemes recognized by the phoneme recognition model is more than that transcribed from the original text, resulting in ***WER***_***Accent*** being greater than one. ***WER***_***Accent*** is not limited to the value range of 0 to 1 as people usually understand, and there is no upper limit to the value range of ***WER***_***Accent***. For example, it cannot be simply said that a ***WER***_***Accent*** value of 0.8 indicates a large degree of accent, but it should be understood that the larger the ***WER***_***Accent***, the larger the degree of accent. However, the relatively small number of such phenomena is related to noise interference and distortion in ATC speech, as well as the data enhancement algorithms of the phoneme recognition model that need to be improved.

**Figure 7:**
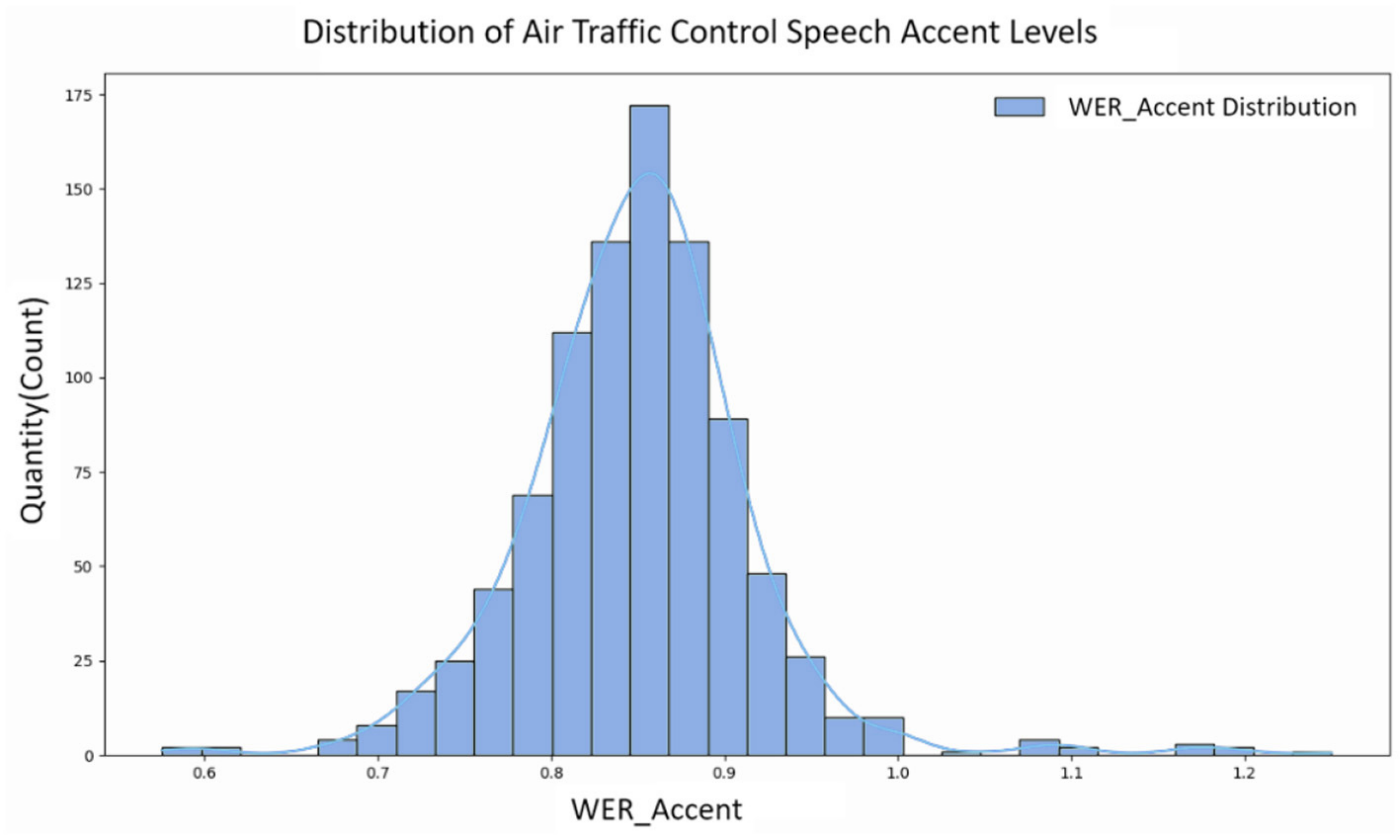
Distribution of degree of ATC speech accent.

Another batch of speeches from the first-line approach control recordings was selected as test data and calculated speech accent degree distribution. According to the calculated speech accent degree distribution, the overall results show a trend of skewed distribution, as shown in Figure 7. Therefore, we artificially divided the ATC speech data tested in the experiment into three different levels based on the quartiles of the speech accent degree data distribution, corresponding to the three accent degree levels of Strong, Medium and Weak, as shown in Table 5. This division allows us to understand the differences in ATC speech accents more clearly and provides a more informative guide for further analyses and applications.

**Table 5:** ATC speech accent level classification and description.

**Level**	**Range**
Weak	[0.576, 0.828)
Medium	[0.828, 0.872)
Strong	[0.872, 1.25)

The ATC speech data tested in this experiment had a significantly skewed right-normal distribution of the degree of accent, with a skewness of 0.7529. The horizontal axis denotes the degree of ATC speech accent, and the vertical axis denotes the number of speech samples at the corresponding degree.

## 4 Experimental impact of different levels of ATC speech accent on speech recognition model accuracy

### 4.1 Speech recognition model accuracy impact evaluation metric

Speech data with different ATC speech accent degree levels were input into different types of ATC speech recognition models to observe their effects on the recognition accuracy of the models. The speech recognition models used in the experiments are Whisper pre-training model, an automatic speech recognition model developed by OpenAI (Radford et al., 2023), and PPASR pre-training model, a speech recognition model developed by Baidu (Zhang et al., 2022), which are publicly available on the web. Both pre-training models were fine-tuned using the same ATC speech dataset in advance before the start of the impact experiments in order to better adapt them to the pronunciation characteristics of ATC speech. Both speech recognition models use the same experimental environment, the same training dataset and test dataset. Although the pre-trained models were publicly downloaded from the web, we chose to pre-train based on the same publicly available dataset. Both pre-training models were fine-tuned using the same ATC speech dataset in advance before the start of the impact experiments in order to better adapt them to the pronunciation characteristics of ATC speech.

The recognition accuracy of each speech recognition model is also calculated using the same principle as in Equation (7), i.e., how many insertion, deletion, and substitution operations need to be performed in order to convert the recognized text output from the model to the original text. The error rate (***WER***_***ATC***_***ASR***) of the ATC speech recognition model calculated here is for the degree of difference between the recognized text and the real text, and the value of (1-***WER***_***ATC***_***ASR***) is used as the accuracy rate of the speech recognition model. After fine-tuning the training, the recognition accuracy of the Whisper-based ATC speech recognition model is 95.07%, and the recognition accuracy of the PPASR-based ATC speech recognition model is 77.21%.

In the process of calculating the correlation between the degree of ATC speech accent and the recognition accuracy of each speech recognition model, since the degree of ATC speech accent is calculated for the degree of difference between the recognized phoneme sequences of the speech to be tested and the real phoneme sequences, the recognition accuracy of the speech recognition model is aimed at the degree of difference between the recognized text and the real text, and also calculates the degree of difference between the two sequences.

Therefore, in order to unify the quantitative metrics, we adopt the minimum edit distance to analyze ATC speech accents and the recognition accuracy of each speech recognition model, which are denoted by the symbols ***Phoneme***_***Edit***_***Distance*** and ***ASR***_***Edit***_***Distance***, respectively. The minimum edit distance is calculated by finding the minimum number of edits required to convert one sequence to another; these edit operations include inserting, deleting, and replacing characters. Minimum edit distance is usually unitless, as it indicates the number of edit operations without involving actual physical or time units. Lower values indicate that the two sequences are more similar; higher values indicate that the two sequences are less similar. Therefore, the larger the edit distance (***Phoneme***_***Edit***_***Distance*** ) between the recognized phoneme sequence and the real phoneme sequence of the audio to be tested, the greater the degree of ATC speech accent; the larger the edit distance (***ASR***_***Edit***_***Distance*** ) between the text recognized by the speech recognition model and the real text, the worse the recognition accuracy. The edit distance calculation formula is shown in Equation (8).


(8) $$D(i,j)\;=\;\min\{\begin{array}{c} D(i-1,j)+1\;\;\;\;\;\;\;\;\;\;(Deletion)\\ D(i,j-1)+1\;\;\;\;\;\;\;\;\;\;(Insertion)\\ D(i-1,j-1)+Cost(S_{i},T_{j})\;(Substitution) \end{array}$$

Where ***D*(*i*, *j*)** denotes the minimum distance required to convert the recognized phoneme sequence ***S*(1:*i*)** to the real phoneme sequence ***T*(1:*j*)** and ***Cost*(*S***_***i***_**, *T***_***j***_**)** is the cost of the substitution, which is 0 if ***S***_***i***_**=*T***_***j***_ and 1 otherwise.

### 4.2 Minimum edit distance per speech in different speech recognition models

**Figure 8:**
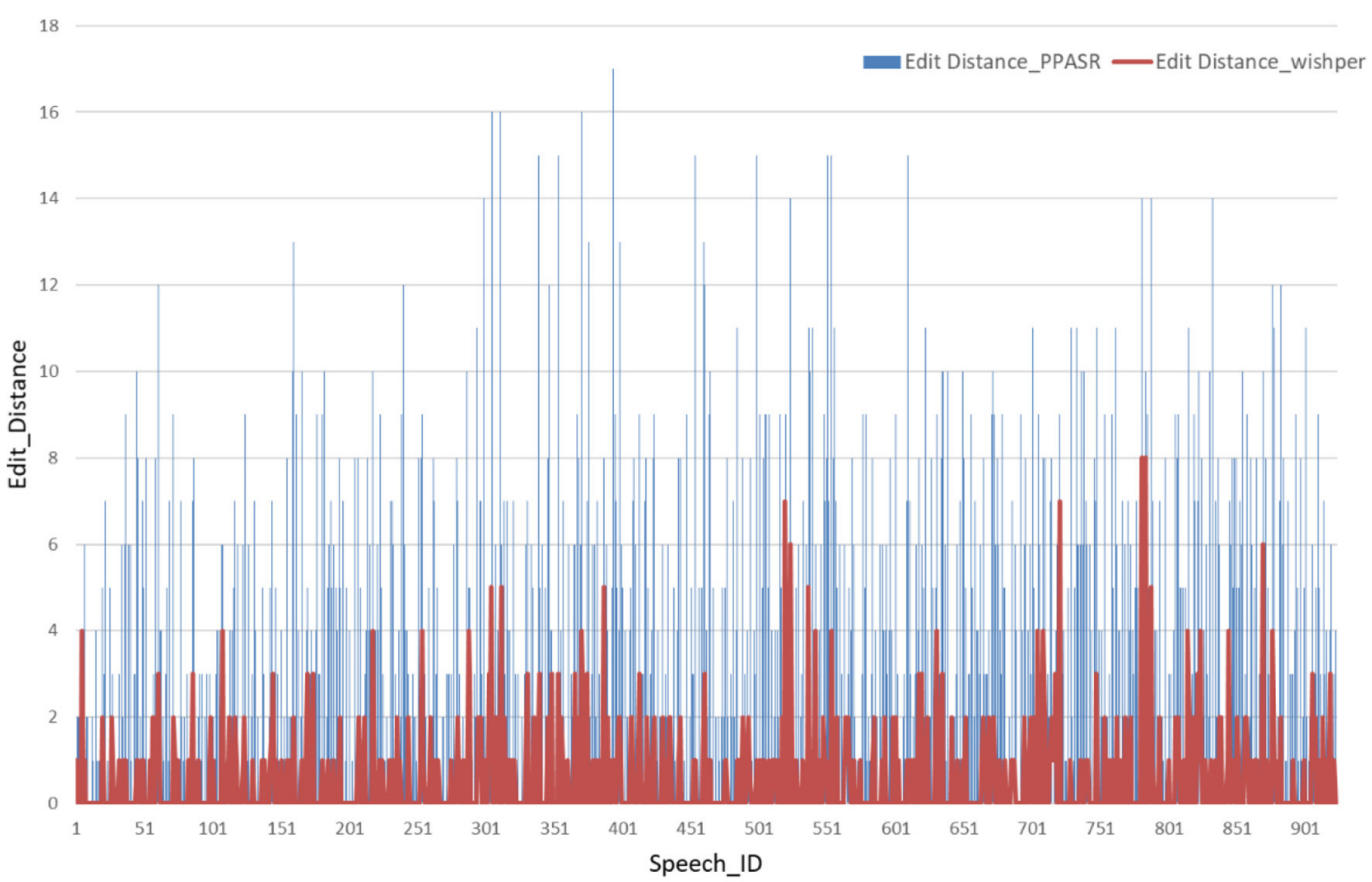
Minimum edit distance for various ATC speech recognition models.

**Figure 9:**
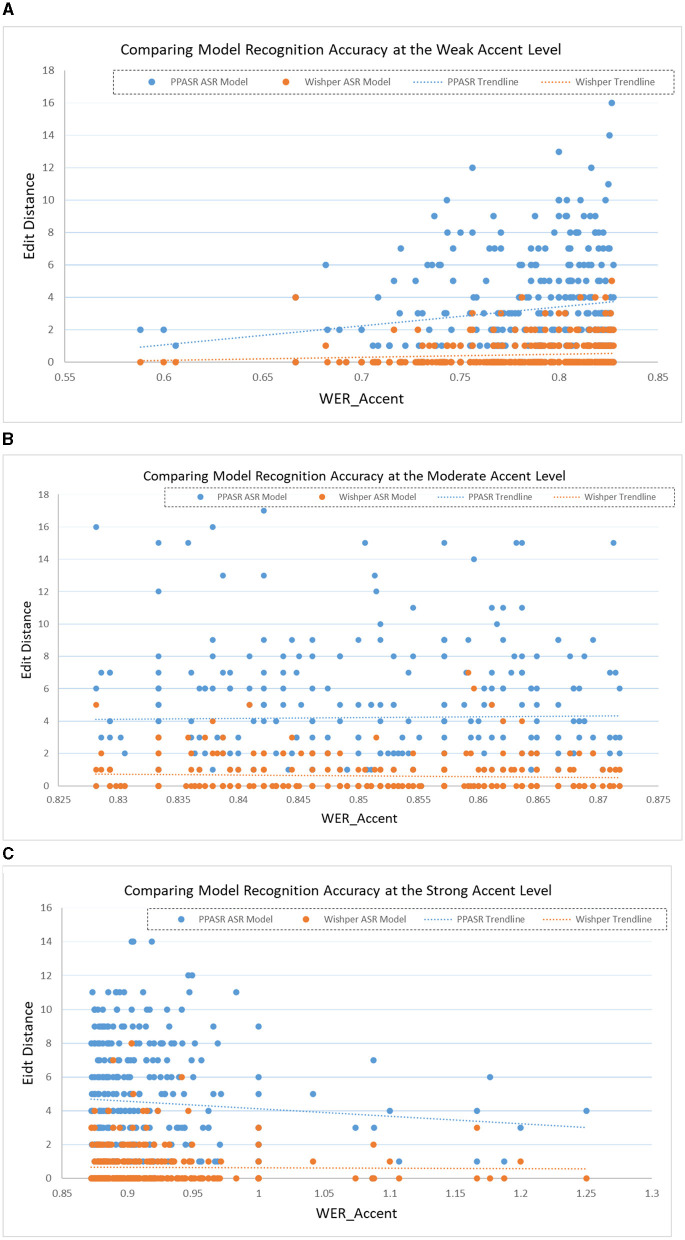
Comparison of various speech recognition model accuracy performance by ATC speech accent levels. **(A)** Comparison of model accuracy performance for weak ATC speech accent level. **(B)** Comparison of model accuracy performance for medium ATC speech accent level. **(C)** Com-parison of model accuracy performance for strong ATC speech accent level.

For experimental validation, we individually selected 1,000 ATC speech from the collected ATC speech corpus as a test set. As shown in Figure 8, the horizontal axis represents the identifier for each speech, such as Speech 1, Speech 2, and so on. The vertical axis corresponds to the minimum edit distance of each speech in the speech recognition model. When confronted with speech data with accents, the minimum edit distance of the Whisper model under each speech is significantly smaller than the performance of the PPASR model. As shown in Figure 9, the minimum edit distance of the Whisper model is also significantly smaller than that of the PPASR model as the degree of accent of the speech on the horizontal axis changes.

In summary, the Whisper-based ATC speech recognition model exhibits higher recognition accuracy than the PPASR-based ATC speech recognition model when faced with ATC speech data of the same accent degree. Similar to the performance of the experimental results in the paper (Radford et al., 2023): the generalization of Whisper as a speech pre-trained large model is better than some company models.

### 4.3 Correlation analysis between ATC speech accents and speech recognition model accuracy

As shown in Table 6, the correlation between ATC speech accents and the recognition accuracy of two distinct speech recognition model is represented by calculating the Pearson correlation coefficients of ***Phoneme***_***Edit***_***Distance*** and ***ASR***_***Edit***_***Distance***.

**Table 6:** The correlation coefficients between different degree of accent in ATC speech and the edit distance of various speech recognition models.

**Level**	**Range**	**Correlation coefficient**	
		**Phoneme_Edit_Distance: Wishper_Edit_Distance**	**Phoneme_Edit_Distance: PPASR_Edit_Distance**
Weak	[0.576, 0.828)	0.02	0.104
Medium	[0.828, 0.872)	0.1498	0.2637
Strong	[0.872, 1.25)	0.0989	0.2573

According to Table 6, it can be seen that for the Whisper-based ATC speech recognition model, the recognition accuracy of the speech recognition model shows a decreasing trend with the deepening of the degree of accent of the speech data, but the correlation is not large; for the PPASR-based ATC speech recognition model, the recognition accuracy of the speech recognition model shows a significant decreasing trend with the deepening of the degree of accent of the speech data, which is slightly larger than the correlation of the Whisper-based ATC speech recognition model.

Therefore, in summary, the accent degree of the ATC speech data used in this experiment can be classified into three levels: strong, medium and weak. Various levels of ATC speech data is input into the ATC speech recognition model based on Wishper and PPASR for speech recognition, and it is found that the recognition accuracy of the speech recognition model decreases with the increase of the accent degree of the speech data. The Wishper-based ATC speech recognition model has a certain correlation with the trend of ATC speech accent level, but the correlation is not as strong as that of the PPASR-based ATC speech recognition model.

Because the two speech recognition models used in this paper are pre-trained models based on the same pre-training set downloaded directly from the web publicly, they are only fine-tuned with the ATC speech data. Therefore, based on the results of this experiment, it can be concluded that the Wishper-based ATC speech recognition model is more robust than the PPASR-based ATC speech recognition model, and is better able to face the challenge of speech containing accent interference. This work also in turn shows in depth that datasets with different accent degree can test the recognition performance of different speech recognition systems or models under accent interference.

Therefore, the experiment designed in this chapter on the effect of different accent degree levels on speech recognition accuracy verifies that different ATC speech accent degree affects the recognition accuracy of ATC speech recognition models, and suggests that accent should be used as one of the metrics for evaluating the quality of ATC speech.

## 5 Conclusion

The traditional speech recognition model development is to distribute all the datasets directly according to a certain ratio without distinguishing the quality of the datasets, which is more random. In future scenarios for the development and testing of ATC speech recognition models, it is essential to introduce data quality assessment methods and adjust dataset allocation in order to effectively address challenges such as the insufficient amount of speech data and difficulties in data acquisition. This approach aims to solve the problem of inadequate model performance caused by uneven data distribution. Specifically, the overall dataset is first divided into levels according to the degree of data quality, and then the appropriate amount of data is selected from various ATC quality to be combined according to the actual application requirements. This approach can manage the data set in a more refined way to ensure the quality and applicability of the data. At the same time, it can provide the required data in a timely manner according to the application requirements, providing strong data support for application performance assurance and testing.

In this study, we have used the mutual integration of deep neural networks and information theory to analyze the effect of accent on the performance of speech recognition models, and constructed an accent phenomenon detection method based on air traffic control speech through deep neural networks. Specifically:

(1) A phoneme recognition model that collocates speech features and sentence a priori text features is built. When the speech to be tested contains an accent, the phoneme recognition model may identify it as a phoneme that does not match the actual phoneme. Therefore, we can assess the degree of speech accent by comparing the differences between the recognized phonemes and the phonemes in the a priori textual transcription of the speech, which enables the quantification of the degree of accent;
(2) In addition to the existing open-source TIMIT data, the data used in this paper are the first-line approach control recordings from the East China Air Traffic Control Bureau of CAAC, which are used as the ATC data set for the experiments, so as to ensure the professionalism of the data;
(3) Using correlation coefficients and comparative experiments, we analyze and compare the accuracy of speech recognition by putting the speech data of various accent degrees into different speech recognition models to analyze the influence of ATC speech accents on the correct recognition rate of the speech recognition models. The speech recognition models used in the experiments are Whisper pre-training model, an automatic speech recognition model developed by OpenAI, and PPASR pre-training model, a speech recognition model developed by Baidu, which are publicly available on the web. Both pre-training models were fine-tuned using the same ATC speech dataset in advance before the start of the impact experiments in order to better adapt them to the pronunciation characteristics of ATC speech.

Therefore, it is shown that, in order to evaluate the quality of ATC speech data more scientifically and finely, the accent can be used as one of the metrics for evaluating the quality of ATC speech. The proposed accent-based speech quality evaluation method, in contrast to the existing manual fuzzy speech quality assessment method, not only reduces human and material resources, but also avoids being susceptible to the influence of subjective factors and individual differences of the evaluator. This greatly improves the objectivity of the evaluation results and the logic of the illustrated speech quality evaluation.

## Data availability statement

The original contributions presented in the study are included in the article/supplementary material, further inquiries can be directed to the corresponding author.

## Author contributions

WP: Conceptualization, Formal analysis, Funding acquisition, Resources, Supervision, Writing—review & editing. JZ: Conceptualization, Data curation, Formal analysis, Investigation, Methodology, Project administration, Software, Validation, Visualization, Writing—original draft. YZ: Data curation, Formal analysis, Investigation, Methodology, Project administration, Writing—review & editing. PJ: Data curation, Software, Validation, Visualization, Writing—review & editing. SH: Formal analysis, Investigation, Resources, Supervision, Writing—review & editing.

## References

[B1] Chan W. Jaitly N. Le Q. V. Vinyals O. (2015). Listen, attend and spell. arXiv Preprint arXiv:1508.01211. 10.48550/arXiv.1508.01211

[B2] Chorowski J. K. Bahdanau D. Serdyuk D. Cho K. Bengio Y. (2015). Attention-Based Models for Speech Recognition. Montréal, QC: Advances in neural information processing systems (NIPS).

[B3] Choudhury S. J. Pal N. R. (2022). Fuzzy clustering of single-view incomplete data using a Multiview framework. IEEE Trans. Fuzzy Syst. 30, 5312–5323. 10.1109/TFUZZ.2022.3173673

[B4] Downs R. R. Ramapriyan H. K. Peng G. Wei Y. (2021). Perspectives on citizen science data quality. Front. Clim. 3, 615032. 10.3389/fclim.2021.615032

[B5] Feng Y. Fu G. Chen Q. Chen K. (2020). SED-MDD: Towards Sentence Dependent end-to-End Mispronunciation Detection and Diagnosis. Barcelona: IEEE International Conference on Acoustics, Speech and Signal Processing (ICASSP).

[B6] Garofolo J. S. (1993). TIMIT Acoustic Phonetic Continuous Speech Corpus. Philadelphia, PA: Linguistic Data Consortium.

[B7] Huang H. Xu H. H. Hu Y. Zhou G. (2017). A transfer learning approach to goodness of pronunciation based automatic mispronunciation detection. J. Acoustical Soc. Am. 142, 3165–3177. 10.1121/1.5011159 29195422

[B8] International Civil Aviation Organization (2009). Manual on the Implementation of ICAO Language Proficiency Requirements. Montreal, QC: ICAO.

[B9] International Telecommunication Union (1996). P.800: Methods for Subjective Determination of Transmission Quality. Geneva: ITU.

[B10] International Telecommunication Union (2001). P. 862: Perceptual Evaluation of Speech Quality (PESQ): An Objective Method for End-to-End Speech Quality Assessment of Narrow-Band Telephone Networks and Speech Codecs. Geneva: ITU.

[B11] Jahchan N. Barbier F. Gita A. D. Khelif K. Delpech E. (2021). Towards an Accent-Robust Approach for ATC Communications Transcription. Brno: Interspeech.

[B12] Kanters S. Cucchiarini C. Strik H. (2009). The Goodness of Pronunciation Algorithm: A Detailed Performance Study. Warwickshire: Speech and Language Technology in Education (SLaTE), Wroxall Abbey Estate.

[B13] Lee A. Chen N. F. Glass J. (2016). Personalized Mispronunciation Detection and Diagnosis Based on Unsupervised Error Pattern Discovery. Shanghai: IEEE International Conference on Acoustics, Speech and Signal Processing (ICASSP).

[B14] Lee A. Glass J. (2012). A Comparison-Based Approach to Mispronunciation Detection. Miami, FL: IEEE Spoken Language Technology Workshop (SLT).

[B15] Lee A. Zhang Y. Glass J. (2013). Mispronunciation Detection via Dynamic Time Warping on Deep Belief Network-Based Posteriorgrams. Vancouver, BC: IEEE International Conference on Acoustics, Speech and Signal Processing(ICASSP).

[B16] Liu Z. (2023). An effective conflict management method based on belief similarity measure and entropy for multi-sensor data fusion. Artificial Intel. Rev. 2023, 1–28. 10.1007/s10462-023-10533-0

[B17] Liu Z. (2024). Fermatean fuzzy similarity measures based on Tanimoto and Sørensen coefficients with applications to pattern classification, medical diagnosis and clustering analysis. Eng. Appl. Artificial Intel. 132, 107878. 10.1016/j.engappai.2024.107878

[B18] Liu Z. Letchmunan S. (2024). Enhanced Fuzzy Clustering for Incomplete Instance with Evidence Combination. ACM Trans. Knowl. Discov. Data. 18, 1–20. 10.1145/3638781

[B19] McAuliffe M. Socolof M. Mihuc S. Wagner M. Sonderegger M. (2017). Montreal Forced Aligner: Trainable Text-Speech Alignment Using Kaldi. Stockholm: Interspeech.

[B20] Pan W. Jiang P. Li Y. Wang Z. Huang J. (2023). Research on automatic pilot repetition generation method based on deep reinforcement learning. Front. Neurorobotics 17, 1285831. 10.3389/fnbot.2023.1285831 PMC10598579 37885770

[B21] Povey D. Ghoshal A. Boulianne G. Burget L. Glembek O. Goel N. Vesely K. (2011). The Kaldi Speech Recognition Toolkit. Madonna di Campiglio: IEEE Workshop on Automatic Speech Recognition and Understanding.

[B22] Radford A. Kim J. W. Xu T. Brockman G. McLeavey C. Sutskever I. (2023). Robust speech recognition via large-scale weak supervision. In: International Conference on Machine Learning. Honolulu, HI: PMLR.

[B23] Romero-Rivas C. Martin C. D. Costa A. (2015). Processing changes when listening to foreign-accented speech. Front. Hum. Neurosci. 9, 167. 10.3389/fnhum.2015.00167 25859209 PMC4373278

[B24] Styler W. (2013). Using Praat for Linguistic Research. Boulder: University of Colorado at Boulder Phonetics Lab.

[B25] Sudhakara S. Ramanathi M. K. Yarra C. Ghosh P. (2019). An improved goodness of pronunciation (GoP) measure for pronunciation evaluation with DNN-HMM system considering HMM transition probabilities. In: International Conference on Spoken Language Processing (INTERSPEECH). Graz, Vienna.

[B26] Tepperman J. Narayanan S. (2008). Using articulatory representations to detect segmental errors in nonnative pronunciation. IEEE Trans. Audio Speech Lang. Proc. 16, 8–22. 10.1109/TASL.2007.909330

[B27] Watanabe S. Hori T. Kim S. Hershey J. R. Hayashi T. (2017). Hybrid CTC/attention architecture for end-to-end speech recognition. IEEE J. Select. Top. Sig. Proc. 11, 1240–1253. 10.1109/JSTSP.2017.2763455

[B28] Witt S. M. (2000). Use of Speech Recognition in Computer-Assisted Language Learning. Doctoral dissertation. Cambridge: University of Cambridge, Location of University.

[B29] Zhang H. Yuan T. Chen J. Li X. Zheng R. Huang Y.. (2022). Paddlespeech: an easy-to-use all-in-one speech toolkit. arXiv Preprint arXiv:2205.12007. 10.18653/v1/2022.naacl-demo.12

